# Supplementary material for: miR-98 Regulates TMPRSS2 Expression in Human Endothelial Cells: Key Implications for COVID-19
Source: Biomedicines. 2020 Oct 30;8(11):462. doi: 10.3390/biomedicines8110462 (PMC7693865; doi:10.3390/biomedicines8110462)
Supplement: Supplementary file 1 [file biomedicines-08-00462-s001.pdf]

Supplementary Material

Supplementary Table S1. Sequences of oligonucleotide primers and product sizes.

	Primer	Sequence (*5′-3′*)	Amplicon (*bp*)
**TMPRSS2**	*Forward*	TAA CTG GTG TGA TGG CGT GT	102
	Reverse	CCA AGG TGA GCA GAG GAG TC	
**GAPDH**	*Forward*	GGC TCC CTT GGG TAT ATG GT	94
	Reverse	TTG ATT TTG GAG GGA TCT CG	

TMPRSS2: Transmembrane protease serine 2; GAPDH: glyceraldehyde 3-phosphate dehydrogenase.

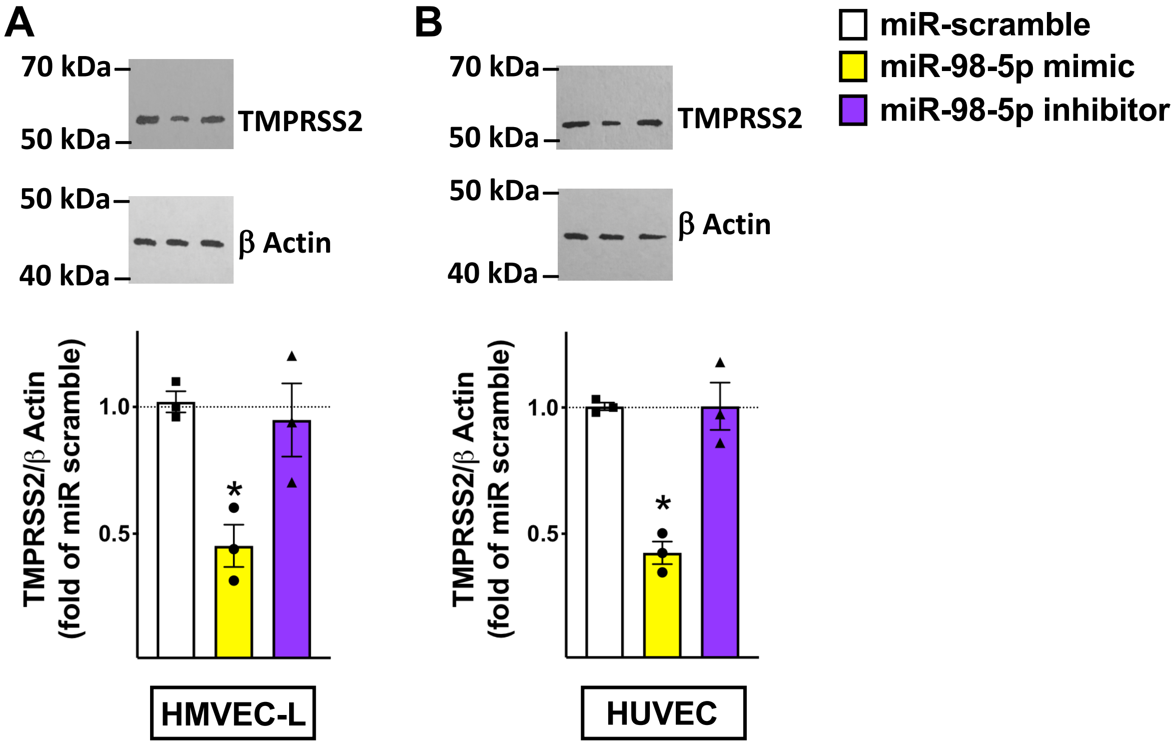

Supplementary Figure S1. TMPRSS2 expression is significantly reduced by miR-98. TMPRSS2 protein levels were assessed by immunoblot in HMVEC-L (**A**) and HUVEC (**B**) lysates 48 hours after transfection with miR-98 mimic, miR-98 inhibitor, and miR-scramble (negative control). Representative immunoblots from three independent experiments are shown. Means ± S.E.M. are shown alongside actual values; *:$p<0.05$.
